# Prediction of brain age using structural magnetic resonance imaging: a comparison of clinical utility of publicly available software packages

**DOI:** 10.1016/j.ebiom.2025.106094

**Published:** 2026-01-02

**Authors:** Ruben P. Dörfel, Brice Ozenne, Melanie Ganz, Jonas E. Svensson, Pontus Plavén-Sigray

**Affiliations:** a Centre for Psychiatry Research, Department of Clinical Neuroscience, Karolinska Institutet, & Stockholm Health Care Services, Region Stockholm, Sweden; b Neurobiology Research Unit, Copenhagen University Hospital Rigshospitalet, Denmark; c Department of Computer Science, University of Copenhagen, Denmark; d Section of Biostatistics, University of Copenhagen, Denmark

**Keywords:** Brain age, Predicted age deviation, Clinical utility, Structural MRI, Machine learning, Biomarker

## Abstract

**Background:**

Brain age estimated from structural magnetic resonance images is commonly used as a biomarker of biological ageing and brain health. Ideally, as a clinically useful biomarker, brain age should indicate the current state of health and be predictive of future disease onset and detrimental changes in brain biology.

**Methods:**

In this preregistered study, we evaluated and compared the clinical utility, i.e., diagnostic and prognostic performance, of six publicly available brain age prediction packages using data from the Alzheimer's Disease Neuroimaging Initiative (ADNI).

**Findings:**

Baseline brain age differed significantly between groups consisting of individuals with normal cognitive function, mild cognitive impairment, and Alzheimer's disease for all packages, but with comparable performance to estimates of grey matter volume. Further, brain age estimates were not centred around zero for participants with normal cognition and showed considerable variation between packages. Finally, brain age was only weakly correlated with disease onset, memory decline, and grey matter atrophy within four years from baseline in individuals without neurodegenerative disease.

**Interpretation:**

The systematic discrepancy between chronological age and brain age among healthy subjects, combined with the weak associations between brain age and longitudinal changes in memory performance or grey matter volume, suggests that the current brain age estimates have limited clinical utility as a biomarker for biological ageing.

**Funding:**

This work was supported by a Longevity Impetus Grant from the Norn Group, the Karolinska Institutet Loo och Hans Ostermans Stiftelse, Gun och Bertil Stohnes Stiftelse, Stiftelsen Gamla Tjänarinnor, Stiftelsen Söderström - Königska and Åhlén-stiftelsen (243016). PPS was supported by a grant from the Swedish 10.13039/501100000942Brain Foundation (PD2024-0444) and the Åke Wibergs Stiftelse (M24-0117).

**Research in context**

**Evidence before this study**

Over the past decade, brain age prediction from structural MRI has become a widely used biomarker of biological ageing and brain health. Existing literature includes numerous studies demonstrating associations between brain age predictions and various health outcomes, cognitive function, and disease risk across different populations. The difference between predicted brain age and chronological age is frequently interpreted as an indicator of accelerated ageing, with researchers applying this metric to answer various clinical research questions across different fields. However, the clinical utility of these predictions has not yet been rigorously evaluated and their usefulness as diagnostic or prognostic biomarkers has recently been called into question.

In this study, we assess the clinical utility of six commonly used brain age prediction models, based on structural MRI scans, that we identified from GitHub, key publications, and PubMed. The selected models employ various architectures, features, and were trained using different datasets, which enabled a robust assessment of the current state of brain age prediction in the field.

**Added value of this study**

Here we assess the clinical utility of brain age model estimates as biomarkers of biological brain ageing. By applying six brain age packages to the Alzheimer's Disease Neuroimaging Initiative dataset, we demonstrate that brain age predictions are only weakly correlated with clinically meaningful outcomes such as memory decline and grey matter atrophy. Notably, brain age estimates consistently failed to outperform simple cerebral grey matter volume measurements in predicting clinical outcomes, highlighting key limitations in current brain age prediction approaches as clinically useful measures of biological ageing.

**Implications of all the available evidence**

Our findings challenge the widespread use of brain age as a biomarker of brain health or biological ageing with implications for research in ageing, psychiatry, and neurodegenerative disorders. The limited clinical utility of current brain age predictions suggests that researchers should exercise caution when interpreting brain age differences as indicators of accelerated, or inhibited, ageing processes. These findings are particularly relevant for clinical trialists considering using brain age model estimates as an endpoint testing neuroprotective or geroprotective interventions.

## Introduction

Biological ageing is commonly defined as the accumulation of molecular and cellular defects that predispose humans to chronic diseases and physical deterioration.^1^^,^^2^ In the brain, these age-related changes manifest as increased atrophy and an accelerated decline in cognitive function.^3^ According to the geroscience hypothesis, ageing-related decline could be delayed or even prevented by pharmacological interventions.^4^ However, prior to conducting clinical trials to test such geroprotective compounds, it is necessary to develop reliable biomarkers that can serve as surrogate endpoints for treatment efficacy.^5^^,^^6^

In the brain, ageing is associated with a range of structural changes. Magnetic resonance imaging (MRI) studies have shown that increasing age is associated with a decline in grey and white matter volume and an enlargement of the ventricles.^7^^,^^8^ The availability of large MRI datasets, combined with advances in machine learning, enabled the development of models to predict the apparent biological age, or *brain age*, of an individual.^9^ Within the last decade, numerous groups have developed algorithms and applied them to clinical data to investigate the potential of brain age as a marker for biological ageing and brain health.^10^ Many researchers have made their trained machine learning models available for public use.^10^, ^11^, ^12^, ^13^, ^14^, ^15^ In a previous study, we compared the test-retest reliability of six publicly available brain age prediction packages and demonstrated that a subset of these packages was both highly accurate and reliable in predicting chronological age.^16^

The primary interest in the framework of *brain age* is not the predicted age itself but rather the deviation from the individual's actual chronological age. This deviation is often referred to as the *predicted age deviation* (PAD). A PAD close to zero suggests that the brain appears typical for its age. Conversely, a positive PAD, where the predicted brain age exceeds the chronological age, suggests that the brain appears older than the norm, indicating that the individual may be ageing faster. In other words, the PAD should be indicative of an individual's ageing trajectory, specifying whether the individual is ageing faster or slower than the norm. As a biomarker of biological ageing, distinct from disease-specific pathology, PAD should capture meaningful variation in ageing-related processes beyond what simpler structural measures provide. Since biological ageing is the primary risk factor, or even driver, for a set of pathological processes in the body and the brain,^4^ the PAD should also be associated with manifest symptoms of such processes, such as atrophy, cognitive decline, and onset of age related diseases.^17^ Therefore, individuals without apparent disease-specific pathology, but with a high PAD (ageing faster), would generally show: 1) accelerated atrophy, 2) accelerated cognitive decline, and 3) accelerated conversion to a neurodegenerative state within the coming years. The current literature presents conflicting evidence regarding the association between PAD and longitudinal changes in brain biology. For example, Elliott et al., 2021^18^ and Gautherot et al., 2021^19^ and longitudinal changes in systemic physiology, cognition, and brain structure, respectively, while results from Vidal-Pineiro et al., 2021^20^ and Korbmacher et al., 2025^21^ found no such correlation.

**Fig. 1:**
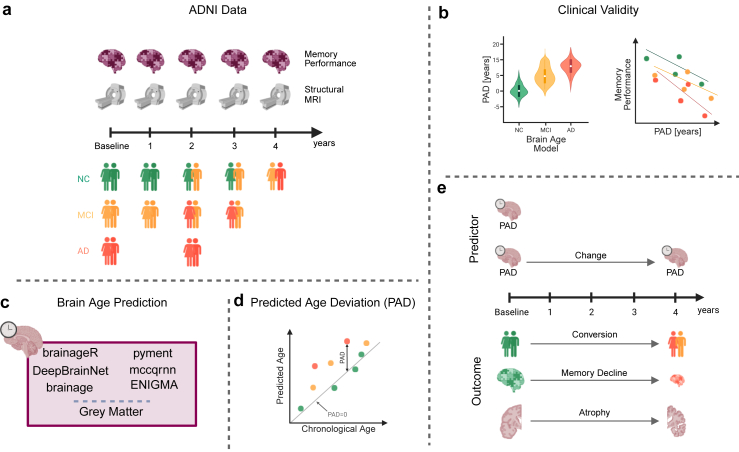
Study overview. **a)** We used data within four years of baseline at one-year intervals. At each time point, memory performance and a structural MR image were available. We had data on participants with normal cognition (NC), mild cognitive impairment (MCI), and Alzheimer's disease (AD). Some participants converted from NC to MCI or AD within the four-year period. **b)** At each time point, we predicted brain age using the six packages (brainageR, DeepBrainNet, brainage, pyment, mccqrnn, and ENIGMA). Additionally, we computed the grey matter volume as a baseline reference. **c)** For each package, we computed the predicted age deviation (PAD) as the difference between the predicted age and chronological age. **d)** In the cross-sectional part of the analysis, we checked for group differences between the groups (NC, MCI, AD) for each package and grey matter. Additionally, we estimated the correlation between the PAD and memory performance at baseline. **e)** In the longitudinal part of the analysis, we first estimated the probability of converting from NC to MCI or AD within four years. Additionally, we computed the correlation between PAD at baseline and memory decline, as well as between PAD at baseline and grey matter atrophy within four years. Finally, we calculated the correlation between changes in PAD and changes in memory performance or grey matter atrophy over a four-year period. Created in BioRender. Rigshospitalet, N. (2026) https://BioRender.com/gi1viw4.

In this study, we evaluated and compared the clinical utility of six popular, pre-trained, ready-to-use brain age prediction packages in both cross-sectional and longitudinal contexts (Fig. 1). We investigated whether the baseline PAD differed between diagnostic groups, whether the PAD was associated with memory function, and whether the PAD was prognostic of neurocognitive disease within four years. Additionally, we investigated whether baseline PAD and changes in PAD over time were associated with the rate of memory decline and grey matter atrophy.

## Methods

### Ethics

Data used in this study was obtained from the Alzheimer's Disease Neuroimaging Initiative (ADNI) database. ADNI was approved by the institutional review boards of all participating sites, and all participants provided written informed consent.

### Study data

We analysed data from 2330 subjects from the ADNI, a longitudinal, multi-site study with comprehensive neuroimaging and neuropsychological testing data. The baseline cohort consisted of participants with normal cognitive function (NC, n = 857, mean age = 72.24 ± 6.63 years), mild cognitive impairment (MCI, n = 1070, mean age = 72.87 ± 7.56 years), and Alzheimer's disease (AD, n = 403, mean age = 74.83 ± 7.80 years). For the longitudinal analyses, we included yearly follow-ups up to four years from baseline. Self-reported demographic characteristics included age, sex, and years of education at baseline. Memory performance was quantified using the ADNI-Mem score, a composite measure derived from memory-related neuropsychological tests.^22^ The score was developed to avoid ceiling effects and to measure changes in memory performance in NC, MCI, and AD groups. Higher scores indicate better memory performance. An overview of the dataset and baseline characteristics is provided in Table 1.

**Table 1:** Participant demographics and scan count by diagnostic group.

Group	Scans [years from baseline]					Baseline				
	0	1	2	3	4	Mean age (SD) [years]	Sex F/M	Mean education (SD) [years]	Mean ADNI-Mem (SD)	Mean AES (SD)
NC	857	464	522	203	284	72.24 (6.63)	487/370	16.53 (2.51)	1.04 (0.58)	2.90 (0.26)
MCI	1070	753	501	237	217	72.87 (7.56)	447/623	15.96 (2.76)	0.19 (0.67)	2.92 (0.25)
AD	403	385	326	120	117	74.83 (7.80)	177/226	15.25 (2.91)	−0.86 (0.54)	3.04 (0.27)
Total	2330	1602	1349	560	618	72.98 (7.33)	1111/1219	16.05 (2.74)	0.32 (0.90)	2.93 (0.26)

This table presents the number of scans acquired at baseline and follow-up visits, along with baseline clinical and demographic measures. Values for age, years of education, memory performance (ADNI-Mem), and average edge strength (AES) are expressed as mean and standard deviation (SD). Sex is reported as the count of females (F) to males (M). The groups include participants with normal cognition (NC), mild cognitive impairment (MCI), Alzheimer's disease (AD), and the total sample.

### Image acquisition, processing, and quality control

All 3D T1-weighted MRI images were acquired at 1.5 T and 3 T. Gradient warping, B1 non-uniformity, and N3 non-uniformity corrected images were downloaded from the ADNI database. These processing steps were applied by the scanner in the ADNI-3 cohort, and by the ADNI team in previous cohorts, so that all images were processed in the same manner. The Clinica pipeline^23^ was used to curate the ADNI data into the Brain Imaging Data Structure.^24^ We then used FreeSurfer^25^ (version 7.2) to derive cortical and subcortical measures for all scans. The intracranial volume and whole-brain grey matter volume were computed using the segmentation pipeline from SPM12.^26^ MRIQC^27^ and FSQC^28^ were used for quality control of raw MR images and FreeSurfer output, respectively. Scans with measures outside the 1.5 interquartile range were flagged for visual inspection. If image artifacts were apparent, the scan was discarded. The average edge strength (AES),^29^ a validated quality metric that reflects motion within MRI scans,^30^ was computed on the brain-masked structural MRI image. AES quantifies the sharpness of tissue boundaries (e.g., grey-white matter), which is important for downstream segmentation and prediction tasks. Lower values indicate better image quality.

### Brain age prediction packages

In line with our previous study,^16^ we used six brain age prediction packages that estimate brain age from structural MRI data: brainageR,^31^ DeepBrainNet,^11^ brainage,^10^ ENIGMA,^14^ pyment,^15^ and mccqrnn.^13^ These packages cover a wide range of different algorithms and input features. For more details, we refer to our previous study^16^ or the original publications.^10^^,^^11^^,^^13^, ^14^, ^15^^,^^31^ Additionally, we used SPM12 to estimate whole-brain grey matter volume normalised by the subject's intracranial volume as a seventh baseline “model”. Grey matter volume typically decreases with age and neurodegeneration,^32^ providing an established anatomical marker of ageing-related brain changes. This serves as a reference point for comparison with the more complex, predictive, brain age models, which should be better predictors of clinically relevant outcomes.^33^

### Statistics

We performed a set of different statistical analyses to assess the clinical utility of the six described brain age prediction packages, preregistered prior to conducting the study (AsPredicted #168021). Deviations from the preregistered analyses and their rationales are detailed in Supplement 1. For all inferential tests, significance levels were set to 0.05, and all p-values and confidence intervals were reported without adjustment for multiple testing. The statistical analysis was performed using R (version 4.4.1), and visualisations were created with Python (version 3.9). The code for the statistical analysis is available online (RDoerfel/bap1b-public).

#### Convergent validity

To evaluate the convergent validity among brain age packages, i.e., the extent to which outcomes intending to measure the same construct are associated with each other, we correlated the baseline PADs for each package with the PADs from the other packages. We reported partial correlation coefficients (PCCs) and their respective 95% confidence intervals (CI).^34^ Higher positive correlations indicate greater convergence between packages. For this analysis, diagnosis (NC, MCI, AD) was included as a covariate to adjust for potential confounding by disease effects on brain structure.

#### Differentiation between clinical groups at baseline

To determine whether brain age models could differentiate between different diagnostic groups, we used a linear model to test for significant differences in the mean PAD between the NC, MCI, and AD groups. We adjusted for age, sex, and AES.^35^^,^^36^ Additionally, we computed effect sizes using *Cohen's d*.

#### Association between memory performance and PAD at baseline

We examined the association between the PAD and memory performance using a linear model with age, sex and AES as covariates.^35^^,^^36^ The association was tested separately for groups of participants with NC, MCI, and AD. We computed PCCs with 95% CI and the corresponding p-values.

#### Association between PAD at baseline and change of diagnostic group

To assess whether the PAD at baseline was associated with the conversion from NC to MCI or AD within four years, we used logistic regression with inverse probability of censoring weighting (IPCW),^37^ accounting for possible non-random study dropout. For this survival analysis, we only included individuals who had NC at baseline. The R package *riskRegression*^38^ was used to compute the IPCW weights with the same covariates as the logistic model, namely age, sex, and AES.^35^^,^^36^ The Brier score and area under the curve (AUC) of the receiver operating characteristic curve were then estimated using a dynamic definition.^39^ To illustrate how the probability of conversion of the IPCW logistic regression varied with PAD or normalised grey matter, we evaluated them for females with mean-centred values for age and AES and varying PAD, or normalised grey matter, at 1 SD below the mean, the mean, and 1 SD above the mean. As a sensitivity analysis, we also applied a Cox proportional hazard regression, and assessed the conversion from MCI to AD.

#### Association between (change in) PAD and longitudinal change in memory performance and grey matter volume

To examine the association between baseline PAD and changes in whole-brain grey matter volume or memory performance over the subsequent four years, we used a linear mixed model to estimate the covariance matrix between these measures. The estimated covariance matrix was then used to calculate the correlations and their 95% CIs. The same approach was applied to estimate the association between changes in PAD and changes in normalised grey matter volume, as well as between changes in PAD and changes in ADNI-Mem. We deviated from the pre-registered analysis and estimated the correlation adjusted on baseline PAD and ADNI-Mem values. The preregistered results are presented in the Supplementary Table S9. We used the 4 × 4 variance-covariance matrix of the two changes and their baseline variables and derived the conditional variance-covariance matrix of the changes given baseline using the standard formula for conditional distributions in multivariate normal theory. The conditional correlation was calculated by dividing the conditional covariance by the product of the square roots of the conditional variances. We used the R packages *mmrm*^40^ and *LMMstar*^41^ for this analysis. As a sensitivity analysis, we added ICV-normalised hippocampal grey matter volume as a dependent variable, since this region has shown stronger volumetric decline in an ageing population compared to that of whole grey matter.^42^

### Role of funders

The funders had no role in the study design, data collection, data analysis, interpretation of results, or writing of the report.

## Results

### Convergent validity

**Fig. 2:**
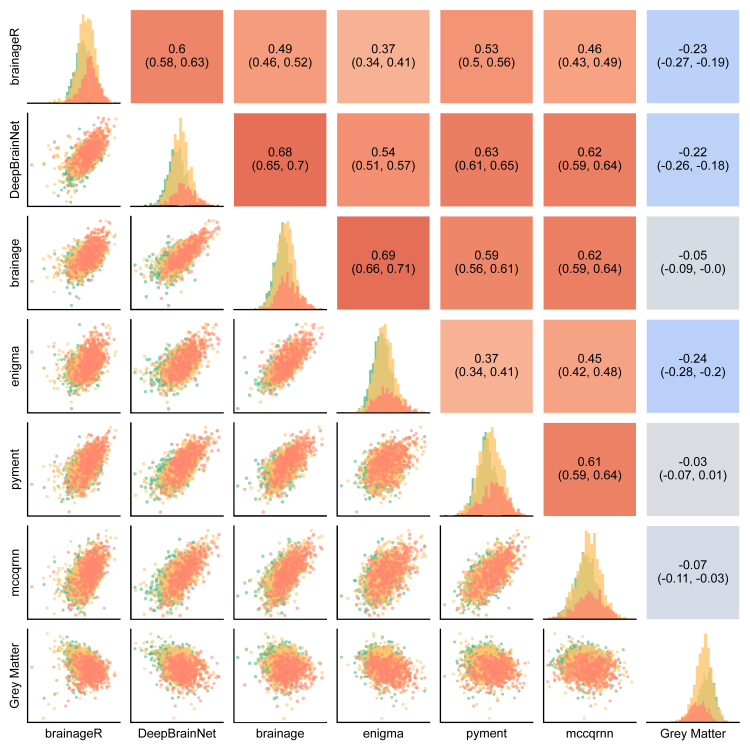
Convergent validity of the PAD across different packages. The scatter plots in the lower triangle visualise the PAD at baseline for each package, colour-coded by diagnosis (NC = green, n = 857; MCI = yellow, n = 1070; AD = red, n = 403). The diagonal presents the histograms, similarly, colour-coded by group. The upper triangle shows the partial correlation coefficient including 95% CI between the two packages, including diagnosis as a covariate.

To assess the extent to which the predictions among packages were correlated with each other, we computed PCCs between the baseline PADs for all packages. Additionally, we computed the correlation of the PAD from each package with the normalised grey matter volume. Group-specific results are presented in Supplementary Table S1. Predictions from all six packages significantly correlated with each other (Fig. 2), with PCCs ranging from 0.37 (95% CI [0.34, 0.41]) between brainageR and ENIGMA, to 0.69 (95% CI [0.66, 0.71]) between brainage and ENIGMA. Further, the correlation between PAD and normalised grey matter volume was considerably weaker, ranging from −0.03 (95% CI [−0.07, −0.01]) for pyment to −0.24 (95% CI [−0.28, −0.20]) for brainageR.

### Differentiation between clinical groups at baseline

**Fig. 3:**
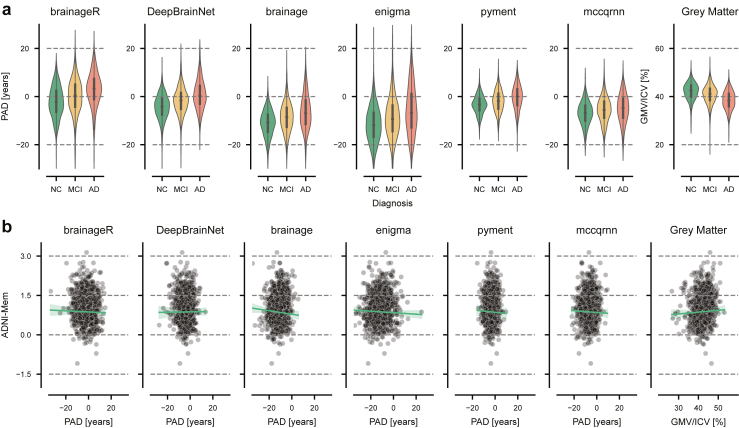
Cross-sectional analysis of the predicted age deviation (PAD). **a)** Comparison of the PAD across packages for participants with normal cognition (NC, n = 857), mild cognitive impairment (MCI, n = 1070), and Alzheimer's disease (AD, n = 403) for six brain age prediction packages and grey matter volume (used as a reference). Grey matter volume (GMV) was normalised by the intracranial volume (ICV). **b)** Baseline PAD vs baseline memory performance (ADNI-Mem) in 857 participants with NC.

We tested for statistical differences in the mean PAD between the diagnostic groups (NC, MCI, AD) using linear models adjusted for age, sex, and image quality. Overall, the mean PAD increased progressively from NC to MCI to AD groups; for example, brainageR showed values of, −2.17 (95% CI [−2.60, −1.74]) years, 0.09 (95% CI [−0.36, 0.53]) years, and 2.80 (95% CI [2.08, 3.53]) years, respectively. However, all models showed a systematic bias, as PAD values for participants with NC were not centred around zero (second column in Table 2). The extended group means are presented in Supplementary Table S2. In addition, predictions had varying degrees of spread across packages, with ENIGMA showing the largest standard deviation in predictions (σ = 7.60 years) and pyment the smallest (σ = 3.77 years) (Fig. 3a). Scatter plots for all groups and packages are presented in Supplementary Figure S1.

**Table 2:** Comparison of predicted age deviation (PAD) and grey matter volume normalised by intracranial volume between groups with normal cognition (NC), mild cognitive impairment (MCI), and Alzheimer's disease (AD).

	NC		NC vs AD			NC vs MCI			MCI vs AD		
	Mean (95% CI)	SD	Beta (SE)	Cohen's d (95% CI)	p-value	Beta (SE)	Cohen's d (95% CI)	p-value	Beta (SE)	Cohen's d (95% CI)	p-value
brainageR	−2.17(−2.6, −1.74)	6.44	4.89 (0.41)	−0.73 (−0.86, −0.61)	<0.001	1.96 (0.32)	−0.32 (−0.41, −0.23)	<0.001	2.61 (0.43)	−0.37 (−0.48, −0.25)	<0.001
Deep BrainNet	−4.33 (−4.67, −3.99)	5.10	5.71 (0.3)	−0.78 (−0.9, −0.66)	<0.001	2.87 (0.23)	−0.45 (−0.54, −0.36)	<0.001	2.92 (0.29)	−0.33 (−0.45, −0.22)	<0.001
brainage	−11.17 (−11.54, −10.8)	5.51	5.31 (0.31)	−0.95 (−1.07, −0.82)	<0.001	2.45 (0.23)	−0.49 (−0.58, −0.40)	<0.001	2.86 (0.28)	−0.45 (−0.56, −0.33)	<0.001
ENIGMA	−11.66 (−12.17, −11.15)	7.60	3.44 (0.24)	−0.71 (−0.83, −0.58)	<0.001	1.50 (0.18)	−0.35 (−0.44, −0.26)	<0.001	1.82 (0.24)	−0.33 (−0.45, −0.22)	<0.001
pyment	−3.38 (−3.63, −3.13)	3.77	6.96 (0.48)	−0.71 (−0.83, −0.59)	<0.001	2.93 (0.35)	−0.35 (−0.44, −0.26)	<0.001	3.96 (0.49)	−0.35 (−0.46, −0.23)	<0.001
mccqrnn	−6.93 (−7.25, −6.6)	4.82	3.04 (0.27)	−0.38 (−0.50, −0.26)	<0.001	1.50 (0.2)	−0.27 (−0.36, −0.18)	<0.001	1.39 (0.26)	−0.11 (−0.23, 0.00)	<0.001
Grey Matter	42.16 (41.91, 42.4)	3.62	−3.30 (0.22)	1.01 (0.89, 1.14)	<0.001	−1.03 (0.16)	0.35 (0.26, 0.44)	<0.001	−2.22 (0.22)	0.62 (0.50, 0.73)	<0.001

For the group with NC, the mean PAD and its standard deviation (SD) are reported as baseline reference values to demonstrate systematic bias across packages. For each comparison between clinical groups (NC vs AD, NC vs MCI, and MCI vs AD), the coefficient (Beta) of the linear model fitted with the covariates age, sex, and AES is reported along with the effect size (Cohen's d) and the p-value. The mean and effect size are reported with their 95% confidence intervals, while the coefficient is reported with its standard error (SE).

In differentiating between groups with NC and AD, we observed the largest effect for the package brainage (Cohen's d = −0.95, 95% CI [−1.07, −0.82]), with participants with AD showing a mean PAD of 5.31 ± 0.31 years (mean ± SE) higher than participants with NC. The smallest effect was found for the package mccqrnn (Cohen's d = −0.38, 95% CI [−0.50, −0.26]), with participants with AD showing a mean PAD 3.04 ± 0.27 years higher than participants with NC.

For the comparison of groups with NC vs MCI, effect sizes were consistently smaller but remained significant across all packages. DeepBrainNet showed the strongest differentiation between groups (Cohen's d = −0.45, 95% CI [−0.54, −0.36]), with participants with MCI having PAD values on average 2.87 ± 0.23 years higher than those of NC individuals. The smallest effect size was observed for the package mccqrnn (Cohen's d = −0.27, 95% CI [−0.36, −0.18]), with participants with MCI showing a mean PAD 1.5 ± 0.20 years higher than those of NC participants.

The differentiation between the groups with MCI and AD was largest for the package brainage (Cohen's d = −0.45, 95% CI [−0.56, −0.33]). Again, mccqrnn showed the weakest effect (Cohen's d = −0.11, 95% CI [−0.23, 0.00]). On average, participants with AD showed higher PAD values than participants with MCI by 2.86 ± 0.28 years for brainage and 1.39 ± 0.26 years for mccqrnn.

For comparison, ICV-normalised grey matter volume showed similar or slightly larger effect sizes than the PAD estimates from brain age packages, particularly between the groups with NC and AD (Cohen's d = 1.01, 95% CI [0.89, 1.14]).

### Association between memory performance and PAD at baseline

We tested for associations between PAD and ADNI-Mem at baseline, hypothesising that an increased PAD would correlate with lower memory performance. We used linear models with age, sex and AES as covariates (Table 3, Fig. 3b, Supplementary Figure S2). The correlations between baseline PAD and memory performance in participants with NC were near zero and non-significant across most brain age prediction packages, with only brainage showing a significant correlation (PCC = −0.07). Correlations ranged from −0.07 (brainage) to 0.01 (DeepBrainNet). However, larger and significant correlations between PAD and memory performance were observed in groups with MCI and AD (all p < 0.001 and all p < 0.039, respectively). In the group with MCI, correlations ranged from −0.33 (DeepBrainNet) to −0.22 (brainageR, mccqrnn), while in the group with AD, correlations ranged from −0.26 (brainage) to −0.10 (mccqrnn). Grey matter volume normalised by intracranial volume showed similar correlations with ADNI-Mem scores as the brain age prediction models across all groups. As a sensitivity analysis, we present the results including education as an additional covariate in Supplementary Table S3. In short, there were only small numerical changes. The correlation for brainage did not remain statistically significant.

**Table 3:** Association between predicted age deviation (PAD) and memory performance (ADNI-Mem) for participants with normal cognition (NC), mild cognitive impairment (MCI), and Alzheimer's disease (AD).

	NC		MCI		AD	
	PCC (95% CI)	p-value	PCC (95% CI)	p-value	PCC (95% CI)	p-value
brainageR	−0.02 (−0.09, 0.04)	0.476	−0.22 (−0.28, −0.16)	<0.001	−0.14 (−0.24, −0.04)	0.005
DeepBrainNet	0.01 (−0.06, 0.07)	0.862	−0.33 (−0.38, −0.27)	<0.001	−0.24 (−0.33, −0.14)	<0.001
brainage	−0.07 (−0.13, −0.0)	0.046	−0.27 (−0.33, −0.21)	<0.001	−0.26 (−0.35, −0.17)	<0.001
ENIGMA	−0.05 (−0.12, 0.01)	0.119	−0.27 (−0.33, −0.22)	<0.001	−0.18 (−0.27, −0.08)	<0.001
pyment	−0.02 (−0.09, 0.04)	0.515	−0.23 (−0.28, −0.17)	<0.001	−0.2 (−0.29, −0.1)	<0.001
mccqrnn	−0.03 (−0.09, 0.04)	0.436	−0.22 (−0.28, −0.16)	<0.001	−0.1 (−0.2, −0.01)	0.037
Grey matter	0.04 (−0.02, 0.11)	0.211	0.18 (0.12, 0.24)	<0.001	0.23 (0.13, 0.32)	<0.001

We report the partial correlation coefficient (PCC) with its 95% confidence interval (CI) and the corresponding p-value between PAD and ADNI-Mem, controlling for the covariates age, sex and image quality. The PCC between ADNI-Mem and grey matter volume normalised by intracranial volume (Grey Matter) is added as a reference.

### Association between PAD at baseline and change of diagnostic group

Among the 861 subjects with NC, 64 converted to MCI or AD within a four-year follow-up period. We investigated whether baseline PAD could predict conversion from NC to MCI or AD within this timeframe using an IPCW logistic regression. The odds ratio (OR) for a one-year increase in PAD or a 1% increase in ICV-normalised grey matter volume varied across methods, with three packages showing significant associations with conversion risk (Table 4). Among the evaluated packages, the strongest association was observed for pyment (OR = 1.16, 95% CI [1.03, 1.29], p = 0.012), followed by brainage (OR = 1.15, 95% CI [1.04, 1.26], p = 0.004). For these two packages, each unit increase in PAD was associated with 15–16% higher odds of conversion from NC to MCI or AD. The remaining packages showed similar effects but did not reach statistical significance. For comparison, normalised grey matter volume showed a significant inverse association with conversion risk (OR = 0.89, 95% CI [0.81, 0.99], p = 0.029).

**Table 4:** Association between predicted age deviation (PAD) and longitudinal disease conversion within four years from baseline.

Model	Odds (95% CI)	p-value	P (x = μ−SD) (95% CI)	P (x = μ) (95% CI)	P (x = μ + SD) (95% CI)	AUC (95% CI)	Brier (95% CI)
brainageR	1.06 (1.0, 1.13)	0.067	0.10 (0.05, 0.19)	0.14 (0.09, 0.21)	0.19 (0.12, 0.29)	0.68 (0.6, 0.77)	0.12 (0.1, 0.15)
DeepBrainNet	1.09 (0.97, 1.23)	0.16	0.09 (0.04, 0.19)	0.13 (0.09, 0.2)	0.19 (0.11, 0.32)	0.69 (0.6, 0.78)	0.12 (0.09, 0.15)
brainage	1.15 (1.04, 1.26)	0.004	0.07 (0.04, 0.13)	0.14 (0.09, 0.2)	0.25 (0.15, 0.4)	0.73 (0.65, 0.81)	0.12 (0.09, 0.14)
ENIGMA	1.07 (1.02, 1.12)	0.007	0.09 (0.05, 0.15)	0.14 (0.09, 0.2)	0.2 (0.13, 0.31)	0.7 (0.62, 0.79)	0.12 (0.09, 0.15)
Pyment	1.16 (1.03, 1.29)	0.012	0.08 (0.04, 0.15)	0.13 (0.09, 0.2)	0.21 (0.13, 0.33)	0.72 (0.64, 0.8)	0.12 (0.09, 0.14)
Mccqrnn	1.05 (0.97, 1.14)	0.189	0.12 (0.07, 0.2)	0.15 (0.1, 0.21)	0.18 (0.11, 0.28)	0.67 (0.59, 0.75)	0.12 (0.1, 0.15)
Grey matter	0.89 (0.81, 0.99)	0.029	0.2 (0.12, 0.3)	0.14 (0.09, 0.21)	0.10 (0.05, 0.18)	0.69 (0.6, 0.77)	0.12 (0.09, 0.15)

The odds ratio and corresponding p-value are given for the coefficient. P(x) provides the probability of converting from normal cognition (NC) to mild cognitive impairment (MCI) or Alzheimer's disease (AD) for a hypothetical individual who has a PAD or grey matter volume (x) at 1 standard deviation (SD) SD below (x = μ – SD) or above (x = μ + SD) the mean (μ) at baseline. In addition, the Brier score and the area under the curve (AUC) are provided as overall performance measures.

**Fig. 4:**
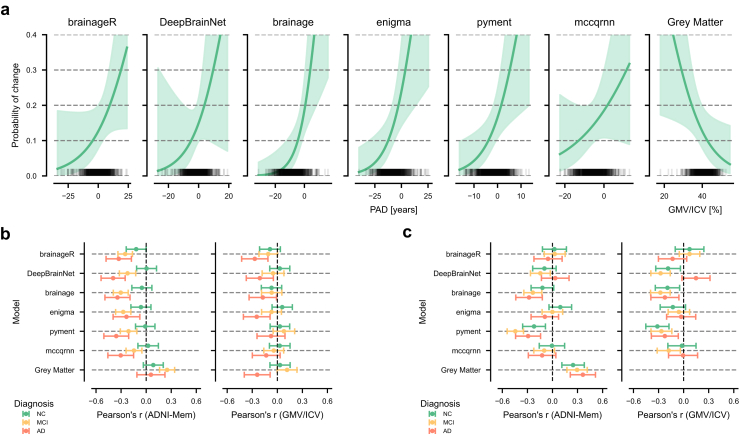
Longitudinal analysis of PAD. **a)** The probability of conversion from normal cognition (NC, n = 857) to mild cognitive impairment (MCI) or Alzheimer's disease (AD) plotted against baseline PAD values for each package. The black rug plot at the base of each subplot shows individual PAD values from the dataset. The curves are estimated with the covariates fixed to their respective means, and sex was set to female. The shaded areas represent the 95% confidence intervals. **b)** The association between baseline PAD and four-year change in normalised grey matter volume (GMV/ICV) and memory performance (ADNI-Mem) by diagnosis (NC = green, n = 857; MCI = yellow, n = 1070; AD = red, n = 403). The error-bars indicate the 95% confidence interval. **c)** The association between four-year change in PAD and four-year change in normalised grey matter volume (GMV/ICV) or memory performance (ADNI-Mem) by diagnosis (NC = green, n = 857; MCI = yellow, n = 1070; AD = red, n = 403). The associations were adjusted for their respective baseline values. The error-bars indicate the 95% confidence interval.

We also estimated conversion probabilities for hypothetical subjects with PAD and grey matter volume at one standard deviation (SD) below the mean, at the mean, and one standard deviation above the mean. These represent population-level probability estimates at different points along the PAD distribution. The general probability curves are presented in Fig. 4a. The conversion probability for having a PAD one SD below the mean ranged from 7 to 12% across methods. In contrast, the conversion probability for having a PAD one SD above the mean increased, ranging from 18 to 25%, with brainage showing the largest difference between conversion probabilities for low and high PAD values (7% vs 25%). Having a mean PAD value resulted in similar conversion probabilities across methods (13–14%). The conversion probability based on grey matter volume followed a similar, but inverted, trend, with conversion probabilities of 20% at one SD below the mean, 14% at the mean, and 10% at one SD above the mean.

The discriminative ability, i.e., the frequency at which individuals with high PAD convert, of the models and grey matter volume was moderate. The AUCs ranged from 0.67 (mccqrnn) to 0.73 (brainage), and their calibration performance was similar across all methods (approximately 0.12).

The results for the MCI to AD conversion are presented in the supplementary materials (Supplementary Table S4 and Supplementary Figure S4). Briefly, both a one-year increase in PAD and a 1% increase in ICV-normalised grey matter volume slightly showed elevated ORs for conversion compared to those observed for NC to MCI/AD conversion across all brain age prediction methods. We also report the results using a traditional Cox proportional-hazards regression, in Supplementary Table S5, with virtually identical results in AUC and Brier score.

### Associations between PAD and rates of decline in grey matter volume and memory function

To further investigate the clinical utility of brain age predictions, we examined the associations between PAD at baseline and subsequent changes in memory performance and normalised grey matter volume over four years across diagnostic groups (Fig. 4b, Supplementary Table S6). Results using change in hippocampal grey matter volume as dependent variable are presented in Supplementary Figure S5a and Supplementary Table S7.

The association between the PAD at baseline and memory decline varied substantially by diagnostic group and model. In individuals with NC, associations with memory decline were small, with brainageR showing the strongest correlation among the packages (r = −0.12, 95% CI [−0.24, 0.00], p = 0.05). In participants with MCI, all models showed significant correlations with future memory decline, ranging from r = −0.30 (brainage) to r = −0.15 (mccqrnn). The strongest associations were observed in participants with AD, where DeepBrainNet achieved the highest correlation (r = −0.40, 95% CI [−0.54, −0.25], p < 0.001), and ENIGMA the smallest (r = −0.24, 95% CI [−0.40, −0.08], p < 0.001).

Baseline PAD showed no significant associations with grey matter atrophy in groups with NC or MCI across all models. In participants with AD, significant but modest correlations were observed for brainageR (r = −0.27, 95% CI [−0.45, −0.09], p = 0.009), ENIGMA (r = −0.25, 95% CI [−0.42, −0.08], p = 0.008), and DeepBrainNet (r = −0.21, 95% CI [−0.39, −0.03], p = 0.026).

To contextualise these findings, we examined baseline normalised grey matter volume as a reference measure. Normalised grey matter volume showed a similar, though inverted, pattern of associations with memory decline compared to the brain age models. In participants with NC, the correlation was r = 0.09 (95% CI [−0.03, 0.20], p = 0.158), in participants with MCI r = 0.25 (95% CI [0.16, 0.34], p < 0.001), and in participants with AD r = 0.06 (95% CI [−0.11, 0.22], p = 0.493). Only the association in participants with MCI was statistically significant. Further, normalised grey matter volume at baseline showed a similar pattern of association with grey matter atrophy compared to brainageR and other models in NC participants with NC, MCI and AD.

### Associations between changes in PAD and rates of decline in grey matter volume and memory function

Further, the analyses from the previous subsection were repeated using the change in PAD within four years instead of baseline PAD to examine the association between longitudinal PAD trajectories and decline in both memory performance and normalised grey matter volume (Fig. 4c, Supplementary Table S8). Changes in PAD and grey matter volume were adjusted for their respective baseline values. The unadjusted results are presented in Supplementary Table S9 and Supplementary Figure S6. Results using change in hippocampal grey matter volume as dependent variable are presented in Supplementary Figures S5b and S7, and Supplementary Table S10.

The association between changes in PAD and changes in memory performance varied markedly across diagnostic groups and models. The strongest associations were found using pyment, which showed significant correlations across all diagnostic groups (NC: r = −0.22, 95% CI [−0.36, −0.08], p = 0.002; MCI: r = −0.44, 95% CI [−0.54, −0.35], p < 0.002; AD: r = −0.29, 95% CI [−0.44, −0.14], p < 0.001). The package brainage also demonstrated consistent negative correlations, particularly in the group with AD (r = −0.28, 95% CI [−0.44, −0.12], p = 0.001) and MCI (r = −0.24, 95% CI [−0.34, −0.13], p < 0.001).

For the association between change in PAD and change in normalised grey matter volume, pyment again demonstrated significant associations across all diagnostic groups (NC: r = −0.31, 95% CI [−0.45, −0.18], p < 0.001; MCI: r = −0.27, 95% CI [−0.39, −0.14], p < 0.001; AD: r = −0.23, 95% CI [−0.39, −0.06], p = 0.009). DeepBrainNet and brainage also showed significant correlations, particularly in the group with MCI (r = −0.28, 95% CI [−0.39, −0.16], p < 0.001 and r = −0.28, 95% CI [−0.4, −0.16], p < 0.001, respectively).

The anatomical reference, which directly quantifies grey matter volume changes, demonstrated significant positive correlations with memory decline across all groups (AD: r = 0.36, 95% CI [0.21, 0.51], p < 0.001; NC: r = 0.25, 95% CI [0.11, 0.38], p = 0.001; MCI: r = 0.29, 95% CI [0.17, 0.42], p < 0.001). As expected, we did not examine the association between change in grey matter volume and change in grey matter volume, as this correlation would be perfect by definition.

## Discussion

Our evaluation of six publicly available brain age prediction packages showed substantial limitations in the clinical utility of current brain age estimates. While the assessed packages demonstrated a modest ability to distinguish between clinical groups and predict disease conversion, we found only moderate convergent validity across different packages and generally only small associations with established markers of brain ageing, such as memory decline and grey matter atrophy. Further, our analysis showed that simple normalised grey matter volume measurements performed comparably to the more complex brain age estimates, putting the added value of existing brain age models as descriptive, diagnostic, or prognostic tools into question.

In recent years, brain age prediction from structural MRI has received great interest, with several research groups making their packages publicly available. Initial results showed that brain age could differentiate between participants who were cognitively unimpaired and participants with neurodegenerative conditions,^10^ and was predictive of mortality,^12^ suggesting that brain age could potentially be developed into a useful biomarker of brain ageing and general health. However, neither the concept nor the packages have been thoroughly evaluated in a comparative setting, and increasing evidence suggests that current brain age estimates are not strongly indicative of the rate of biological ageing of a person.^20^^,^^21^ Here, we expand on our previous comparative reliability assessment of brain age prediction packages^16^ by investigating their clinical utility using a large longitudinal dataset that includes individuals with normal cognition and neurodegenerative diseases.

Brain age prediction packages vary in their preprocessing steps (such as registration to a common space, segmentation, surface-based analysis), the model architecture (statistical learning vs deep learning), the type of input features (voxel-based vs regional, whole-brain or restricted to grey matter), and the distribution of the training data (age, sex, ethnicity). Despite this heterogeneity, the outcome of such packages is commonly referred to as “brain age”, without much distinction. Here, we demonstrate that there is only moderate convergent validity (i.e., agreement) between packages, with the largest partial correlation (adjusting for diagnosis) being r = 0.69 (95% CI [0.66, 0.71]). In this case, if a healthy individual has a PAD of +10 years from ENIGMA, the corresponding 95% prediction interval suggests their residual PAD from the package brainage could range from −9.28 to +8.31 years, spanning 17.6 years. This limited agreement is further exemplified by the large variability across packages (Fig. 3a). For instance, a PAD of ten years may fall within normal variation when calculated using ENIGMA but could indicate significant deviation from the norm when derived from pyment. However, some of the tested models use different input features, such as regional grey matter volume or 3D MRI volumes. The different packages could therefore capture slightly different neurobiological processes. A moderate convergence is therefore in line with comparisons of various biological ageing clocks.^43^

Brain age prediction aims to quantify deviations from population norms, where a PAD of zero is expected to indicate age-appropriate brain health. However, our analysis demonstrates that while PADs correctly rank clinical groups by disease severity, they are significantly biased (i.e., exhibit a non-zero mean) and vary considerably across packages (Fig. 3a). Except for brainageR, most packages estimate PADs for the group with AD that are centred at or below zero. In such cases, a PAD of zero would not indicate a healthy brain but instead suggest an advanced neurodegenerative state. Therefore, a PAD of zero holds little intrinsic meaning, and values must be interpreted in relation to the specific model and sample context rather than as an absolute indicator of deviation from the norm. This bias is a well-documented and discussed limitation in the field of brain age estimation, and likely originates from model weights being affected by regression toward the mean age of the training population.^16^^,^^44^ It has thus been recommended to include age as a covariate when testing for statistical differences to account for this bias.^44^

It has previously been suggested that, as ageing biomarkers more accurately predict age, they become less clinically useful.^45^^,^^46^ In other words, brain age models that are highly accurate in predicting chronological age do not capture biologically meaningful variance in their PADs. However, others have raised concerns that, for more loosely fitting models, PADs are confounded by epistemic uncertainty which makes interpretation of PAD as absolute units difficult.^47^ In this study, we found no clear performance advantage for either the more accurate models or the more loosely fitting models.

There was no significant association between PAD and memory performance in individuals with NC. While PAD correlated with decreased memory performance in groups with MCI and AD for many of the evaluated packages, it should ideally also be indicative of memory performance in individuals with no known neurological conditions, assuming it does reflect the health state of the brain.

PAD at baseline in individuals with NC was associated with conversion to MCI or AD. However, the AUC for the different packages (67%–73%) was comparable to that of normalised grey matter volume (69%). Further, the ROC curve (see Supplementary Figure S3) of a simple model including only the covariates age, sex, and AES was comparable to those including grey matter volume or the different brain age predictions, suggesting little additional prognostic value from brain age models or grey matter volume in individuals with NC. Importantly, the low number of conversions from NC to MCI/AD leads to large CIs with widths up to 0.2 on the AUC scale. More data is needed to precisely characterise the AUC of the brain age models. Further, our reported performance metrics likely represent an upper bound of the out-of-sample performance since we did not compute the AUC on a hold-out validation set. We expect the difference to out-of-sample estimates to be minor,^48^ since the number of parameters in the predictive model was small compared to the number of observations and events (4, 857, and 65, respectively). However, PAD at baseline demonstrated slightly improved prediction of conversion in participants with MCI compared to NC, indicating some added prognostic value in populations already experiencing cognitive impairment.

PAD at baseline was not significantly associated with memory decline or grey matter atrophy within the four-year follow-up period in individuals with NC. If brain age is to function as an efficient marker of biological ageing, we would expect individuals with an increased PAD–those who supposedly age faster–to show accelerated memory decline and grey matter atrophy. In the groups with MCI and AD, the associations were larger and statistically significant, consistent with the assumption that these subjects are on a neurodegenerative trajectory. Additionally, the correlation in participants with AD between normalised grey matter volume at baseline and subsequent atrophy was negative. This trend likely reflects that individuals with severe AD and pronounced baseline atrophy have less remaining volume to lose over time, whereas affected individuals with greater baseline grey matter volume are positioned to experience larger future losses. However, the weak, non-significant associations between the PAD at baseline and a decline in grey matter volume or memory performance in individuals with NC raise questions about the utility of brain age estimation as a proxy for brain ageing rates or trajectories in non-diseased populations. These results align well with data reported by Vidal-Pineiro et al., 2021^20^ and Korbmacher et al., 2025,^21^ who showed a similar non-significant trend in individuals with NC for grey matter atrophy. Of note, for the pyment package, changes in PAD were significantly associated with changes in grey matter volume or memory performance across all groups. Further, an important consideration is that while our statistical models assume linear trends due to the few timepoints available, brain ageing and dementia-related changes likely follow nonlinear trajectories.^49^, ^50^, ^51^ Individuals may be at different stages of brain ageing at baseline, where subsequent volume loss or brain age acceleration may slow down or accelerate. However, our observation of significant correlations between baseline PAD and subsequent changes in grey matter volume in groups with MCI and AD suggests that such ceiling/floor effects do not limit our results. As suggested by Smith et al., 2025^52^ it is difficult, if not impossible, to disentangle variation in PAD caused by biological ageing, measurement noise, and congenital factors when using cross-sectional data. They provide a theoretical explanation for why the conventional framework of predicting chronological age from structural brain features shows only limited association with ageing-related change. Here, we demonstrate that this limitation persists across a heterogeneous set of prediction packages. A potential solution could be to focus on longitudinal changes in brain structure, rather than relying solely on cross-sectional data, in future development of brain age models.^53^^,^^54^

It is important to note that brain age is not intended to serve as a biomarker of AD pathology or disease progression and should not be expected to compete with established AD biomarkers in predicting disease onset or trajectory. However, ageing, characterised by the accumulation of molecular and cellular damage, represents a primary risk factor for AD and other neurodegenerative diseases.^2^^,^^3^ Therefore, if brain age captures meaningful biological ageing processes in the brain, we would expect it to show some association with subsequent AD onset in individuals with NC, particularly when assessed prior to clinical symptoms. Such an association would not reflect brain age's utility as an AD-specific biomarker, but rather its utility as a marker of biological ageing processes that influence vulnerability to age-related neurodegeneration.

Cerebral grey matter volume showed comparable performance to more complex brain age prediction models across all our analyses. Instead of using a constructed biomarker of ageing, alternative approaches using direct grey matter volume estimates might offer more clinically interpretable metrics, especially since more grey matter is generally perceived as being advantageous, while accelerated atrophy is indicative of neurodegeneration and has been linked to adverse clinical outcomes.^53^^,^^55^ As such, grey matter volume has been used as an endpoint in clinical trials evaluating putative neuroprotective compounds.^56^ Normative models of structural decline provide one such approach that enables an explicit assessment of deviations from the population,^7^ whereas brain age, as a concept, only captures this implicitly.

Several limitations of our study should be noted. We focused on a restricted follow-up period (four years) for the longitudinal analyses. Especially in individuals with NC, a longer time interval might be necessary to detect meaningful changes in grey matter volume^57^ and memory performance. However, for brain age to be useful as a potential surrogate biomarker in clinical trials of, e.g., neuroprotective or preventive interventions, it should ideally be able to detect changes within a few years. Additionally, we acknowledge that measurement noise in longitudinal data could introduce regression dilution bias, potentially attenuating the observed associations toward the null. While we cannot rule out such an effect, substantial regression dilution bias in these widely-used packages would itself suggest limitations in their utility. We also selected to focus specifically on memory function by using the ADNI-Mem composite score. Hence, the lack of significant associations between ADNI-Mem and PAD estimates cannot be extrapolated to cognition in general but is restricted to memory performance. The score itself was not age-corrected, meaning that any association between PAD or grey matter volume with ADNI-Mem in the longitudinal analysis could be confounded by age. Further, we only included a subset of existing brain age estimation packages in this analysis, as new packages are developed and published on a regular basis. We, therefore, cannot claim to provide a systematic comparison of the clinical utility of all existing brain age models in the field. Still, the implemented packages offer a wide range of preprocessing pipelines, input features, and model architectures. Since no package consistently outperformed the others, we suggest that more fundamental considerations may need to be addressed, such as the use of cross-sectional data in general,^52^ rather than the choice of model architecture and input features. We also did not retrain any of the algorithms on similar training data to make a full head-to-head comparison of the different model architectures. Instead, we compared the clinical utility of existing publicly available packages when used as “off-the-shelf” estimation methods. Finally, the ADNI cohort is not representative of the broader clinical population, being biased toward highly educated and white individuals and heavily enriched for Alzheimer's disease. Therefore, these clinical utility findings may not generalise to real-world settings. Future studies should aim to validate these results in more diverse, population-representative cohorts.

Our study focused on specific clinical outcomes (disease conversion, memory decline, and grey matter atrophy). While current brain age models showed limited utility for these outcomes in populations with normal cognition, other applications were not evaluated in this study and may warrant further investigation. For instance, it remains possible that brain age markers might prove more useful for predicting broader, more distal outcomes such as quality of life or general physical functioning in older age, or for stratifying research cohorts. However, the substantial variation in PAD estimates across packages, combined with their weak associations with established markers of brain ageing and other limitations discussed elsewhere,^20^^,^^52^ warrant caution when interpreting brain age results, especially when estimates are derived from different packages. One alternative for future development of image-based ageing biomarkers could be to focus on modelling longitudinal trajectories rather than relying solely on cross-sectional data^54^^,^^58^ and incorporating relevant clinical outcomes already in the training process.

In conclusion, the brain age framework has previously shown promise as a potential biomarker for biological brain ageing, primarily through its application of non-linear models to large imaging datasets that account for more complex patterns of brain ageing.^10^, ^11^, ^12^, ^13^, ^14^, ^15^ However, in this study, we show that baseline PAD performs similarly to grey matter volume in terms of prognostic performance and PAD does not strongly correlate with clinical outcomes, such as memory decline or grey matter atrophy, in individuals with NC, and only modestly so in clinical populations. This suggests that the current brain age estimates have limited clinical utility as a biomarker for biological brain ageing.

## Contributors

Conceptualisation: RPD, JES, PPS.

Data curation: ADNI, RPD.

Formal analysis: RPD, BO, MG, JES, PPS.

Funding acquisition: JES, PPS.

Methodology: RPD, BO, MG.

Supervision: BO, MG, JES, PPS.

Visualisation: RPD.

Writing—original draft: RPD.

Writing—review & editing: RPD, BO, MG, JES, PPS.

All authors read and approved the final version of the manuscript. RPD and PPS accessed and verified the underlying data. The ADNI was responsible for patient recruitment, data acquisition, and data sharing.

## Data sharing statement

Sharing participant-level data is prohibited under the ADNI Data Usage Agreement. The authors are happy to provide additional group-level data upon request. The source code for the statistical analysis to reproduce the figures and tables is available here: https://github.com/RDoerfel/bap1b-public. Requests for summary statistics and group-level statistics should go to RPD (ruben.dorfel@ki.se). To access participant-level data, please refer to ADNI (https://ida.loni.usc.edu/collaboration/access/appLicense.jsp).

## Declaration of interests

The authors declare no competing interests.
