# Supplementary material for: Prediction of brain age using structural magnetic resonance imaging: a comparison of clinical utility of publicly available software packages
Source: eBioMedicine. 2026 Jan 2;123:106094. doi: 10.1016/j.ebiom.2025.106094 (PMC12805294; doi:10.1016/j.ebiom.2025.106094)
Supplement: Supplementary material [file mmc1.docx]

Prediction of brain age using structural magnetic resonance imaging: A comparison of clinical utility of publicly available software packages
--
Supplementary Material

# Supplement 1 – Deviations from the Preregistration

We deviated from the preregistration by including an analysis to assess convergent validity between measurement packages, which was not originally planned. We were uniquely positioned to assess this convergent validity as we had predictions available from all measurement packages. Further, following recommendations during the review process, we are adjusting for the baseline values of PAD and grey matter volume or ADNI_MEM to ensure that the correlation was not confounded by initial differences. The unadjusted, pre-registered results are reported in Supplementary Table 9.

# Supplement 2 – Extended Figures and Results

### Convergent Validity

| **Supplementary Table 1:** Correlations between the predicted age deviation (PAD) across packages for participants with normal cognition (NC), mild cognitively impaired (MCI), and Alzheimer’s Disease (AD). | | | | | | | | | | | | | | |
| --- | --- | --- | --- | --- | --- | --- | --- | --- | --- | --- | --- | --- | --- | --- |
|  |  | **brainageR** | **DeepBrainNet** | | | **brainage** | **enigma** | | **pyment** | | **mccqrnn** | | **Grey Matter** | |
| NC | **brainageR** | 1.0 (1.0, 1.0) | | 0.6 (0.55, 0.64) | 0.41 (0.35, 0.46) | | | 0.33 (0.27, 0.39) | | 0.45 (0.4, 0.5) | | 0.44 (0.38, 0.49) | | -0.24 (-0.3, -0.18) |
|  | **DeepBrainNet** | 0.6 (0.55, 0.64) | | 1.0 (1.0, 1.0) | 0.54 (0.49, 0.58) | | | 0.44 (0.38, 0.49) | | 0.52 (0.46, 0.56) | | 0.52 (0.47, 0.57) | | -0.3 (-0.36, -0.24) |
|  | **brainage** | 0.41 (0.35, 0.46) | | 0.54 (0.49, 0.58) | 1.0 (1.0, 1.0) | | | 0.71 (0.68, 0.74) | | 0.45 (0.39, 0.5) | | 0.53 (0.48, 0.58) | | -0.1 (-0.16, -0.03) |
|  | **enigma** | 0.33 (0.27, 0.39) | | 0.44 (0.38, 0.49) | 0.71 (0.68, 0.74) | | | 1.0 (1.0, 1.0) | | 0.29 (0.23, 0.35) | | 0.38 (0.32, 0.43) | | -0.19 (-0.25, -0.12) |
|  | **pyment** | 0.45 (0.4, 0.5) | | 0.52 (0.46, 0.56) | 0.45 (0.39, 0.5) | | | 0.29 (0.23, 0.35) | | 1.0 (1.0, 1.0) | | 0.53 (0.48, 0.58) | | -0.06 (-0.13, 0.0) |
|  | **mccqrnn** | 0.44 (0.38, 0.49) | | 0.52 (0.47, 0.57) | 0.53 (0.48, 0.58) | | | 0.38 (0.32, 0.43) | | 0.53 (0.48, 0.58) | | 1.0 (1.0, 1.0) | | -0.11 (-0.18, -0.04) |
|  | **Grey Matter** | -0.24 (-0.3, -0.18) | | -0.3 (-0.36, -0.24) | -0.1 (-0.16, -0.03) | | | -0.19 (-0.25, -0.12) | | -0.06 (-0.13, 0.0) | | -0.11 (-0.18, -0.04) | | 1.0 (1.0, 1.0) |
| MCI | **brainageR** | 1.0 (1.0, 1.0) | | 0.61 (0.57, 0.64) | 0.5 (0.45, 0.54) | | | 0.39 (0.34, 0.44) | | 0.54 (0.5, 0.58) | | 0.45 (0.4, 0.5) | | -0.28 (-0.34, -0.23) |
|  | **DeepBrainNet** | 0.61 (0.57, 0.64) | | 1.0 (1.0, 1.0) | 0.7 (0.67, 0.73) | | | 0.54 (0.5, 0.58) | | 0.66 (0.62, 0.69) | | 0.63 (0.59, 0.66) | | -0.21 (-0.27, -0.15) |
|  | **brainage** | 0.5 (0.45, 0.54) | | 0.7 (0.67, 0.73) | 1.0 (1.0, 1.0) | | | 0.68 (0.64, 0.71) | | 0.6 (0.56, 0.64) | | 0.63 (0.6, 0.67) | | -0.04 (-0.1, 0.02) |
|  | **enigma** | 0.39 (0.34, 0.44) | | 0.54 (0.5, 0.58) | 0.68 (0.64, 0.71) | | | 1.0 (1.0, 1.0) | | 0.37 (0.32, 0.42) | | 0.45 (0.41, 0.5) | | -0.24 (-0.3, -0.19) |
|  | **pyment** | 0.54 (0.5, 0.58) | | 0.66 (0.62, 0.69) | 0.6 (0.56, 0.64) | | | 0.37 (0.32, 0.42) | | 1.0 (1.0, 1.0) | | 0.63 (0.6, 0.67) | | -0.04 (-0.1, 0.02) |
|  | **mccqrnn** | 0.45 (0.4, 0.5) | | 0.63 (0.59, 0.66) | 0.63 (0.6, 0.67) | | | 0.45 (0.41, 0.5) | | 0.63 (0.6, 0.67) | | 1.0 (1.0, 1.0) | | -0.05 (-0.11, 0.01) |
|  | **Grey Matter** | -0.28 (-0.34, -0.23) | | -0.21 (-0.27, -0.15) | -0.04 (-0.1, 0.02) | | | -0.24 (-0.3, -0.19) | | -0.04 (-0.1, 0.02) | | -0.05 (-0.11, 0.01) | | 1.0 (1.0, 1.0) |
| AD | **brainageR** | 1.0 (1.0, 1.0) | | 0.61 (0.54, 0.67) | 0.59 (0.52, 0.65) | | | 0.4 (0.31, 0.48) | | 0.62 (0.55, 0.68) | | 0.52 (0.45, 0.59) | | -0.07 (-0.17, 0.02) |
|  | **DeepBrainNet** | 0.61 (0.54, 0.67) | | 1.0 (1.0, 1.0) | 0.82 (0.79, 0.85) | | | 0.68 (0.63, 0.73) | | 0.73 (0.68, 0.77) | | 0.73 (0.68, 0.77) | | -0.14 (-0.23, -0.04) |
|  | **brainage** | 0.59 (0.52, 0.65) | | 0.82 (0.79, 0.85) | 1.0 (1.0, 1.0) | | | 0.67 (0.61, 0.72) | | 0.73 (0.68, 0.77) | | 0.7 (0.65, 0.75) | | 0.02 (-0.08, 0.12) |
|  | **enigma** | 0.4 (0.31, 0.48) | | 0.68 (0.63, 0.73) | 0.67 (0.61, 0.72) | | | 1.0 (1.0, 1.0) | | 0.47 (0.39, 0.54) | | 0.54 (0.47, 0.61) | | -0.3 (-0.38, -0.21) |
|  | **pyment** | 0.62 (0.55, 0.68) | | 0.73 (0.68, 0.77) | 0.73 (0.68, 0.77) | | | 0.47 (0.39, 0.54) | | 1.0 (1.0, 1.0) | | 0.68 (0.62, 0.73) | | 0.02 (-0.08, 0.12) |
|  | **mccqrnn** | 0.52 (0.45, 0.59) | | 0.73 (0.68, 0.77) | 0.7 (0.65, 0.75) | | | 0.54 (0.47, 0.61) | | 0.68 (0.62, 0.73) | | 1.0 (1.0, 1.0) | | -0.06 (-0.15, 0.04) |
|  | **Grey Matter** | -0.07 (-0.17, 0.02) | | -0.14 (-0.23, -0.04) | 0.02 (-0.08, 0.12) | | | -0.3 (-0.38, -0.21) | | 0.02 (-0.08, 0.12) | | -0.06 (-0.15, 0.04) | | 1.0 (1.0, 1.0) |

### Differentiation between clinical groups at baseline

| **Supplementary Table 2:** The mean and 95% confidence interval (CI) of the predicted age deviation (PAD) or normalised grey matter volume for each of the three diagnostic groups: normal cognition (NC), mild cognitive impairment (MCI), Alzheimer’s disease (AD). | | | |
| --- | --- | --- | --- |
| **Diagnosis** | **NC** **mean (95%CI) [years]** | **MCI** **mean (95%CI) [years]** | **AD** **mean (95%CI) [years]** |
| **brainageR** | -2.17 (-2.60, -1.74) | 0.09 (-0.36, 0.53) | 2.8 (2.08, 3.53) |
| **DeepBrainNet** | -4.33 (-4.67, -3.99) | -1.72 (-2.05, -1.38) | 0.83 (0.23, 1.43) |
| **brainage** | -11.17 (-11.54, -10.80) | -8.55 (-8.92, -8.18) | -6.37 (-7.10, -5.65) |
| **enigma** | -11.66 (-12.17, -11.15) | -8.78 (-9.29, -8.26) | -5.62 (-6.62, -4.63) |
| **pyment** | -3.38 (-3.63, -3.13) | -1.93 (-2.20, -1.66) | -0.39 (-0.89, 0.10) |
| **mccqrnn** | -6.93 (-7.25, -6.60) | -5.55 (-5.86, -5.23) | -4.94 (-5.53, -4.35) |
| **Grey Matter** | 42.16 (41.91, 42.40) | 40.83 (40.59, 41.06) | 38.42 (38.04, 38.79) |

| 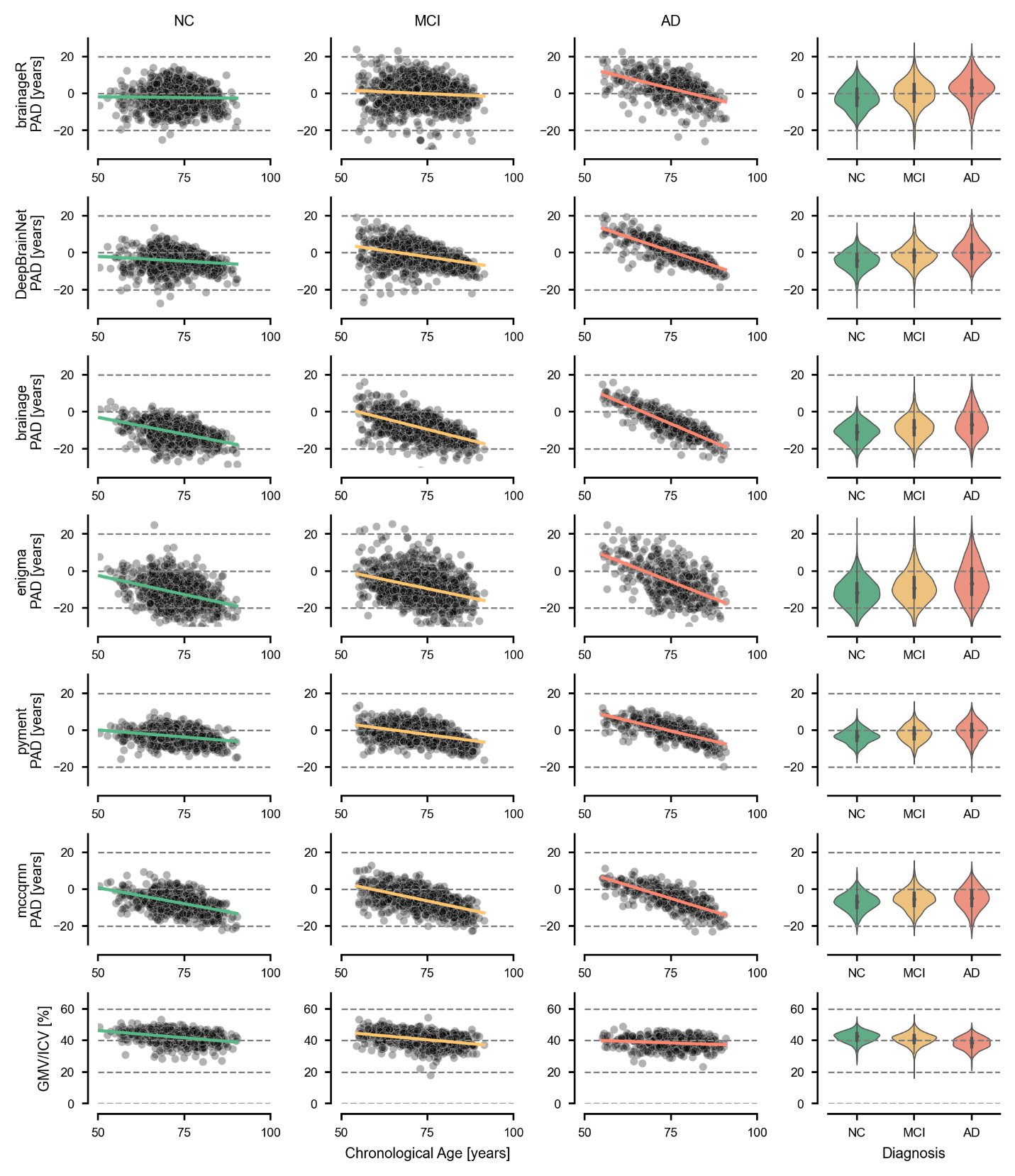 |
| --- |
| **Supplementary Figure 1:** PAD and grey matter volume across diagnostic groups. Cross sectional comparison of the predicted age deviation (PAD) across packages for participants with normal cognition (NC), mild cognitive impairment (MCI), and Alzheimer’s disease (AD) for six brain age prediction packages and grey matter volume (used as a reference). |

### Association between memory performance and PAD at baseline

We added years of education as a covariate when ADNI-Mem was the outcome of interest, given education's strong association with memory-related measures and potential for confounding ^1^. For interpretability, we report partial correlations between ADNI-MEM and PAD controlling for years of education, AES, age, and sex ^2^.

| **Supplementary Table 3:** Association between PAD and memory performance (ADNI-Mem) for participants with normal cognition (NC), mild cognitively impaired (MCI), and Alzheimer’s disease (AD). We report the partial correlation coefficient (PCC) with its 95% confidence interval (CI) and the corresponding p-value between PAD and ADNI-Mem, controlling for the covariates age, sex, AES, and years of education. The PCC between ADNI-Mem and grey matter volume normalised by intracranial volume is added as a reference. | | | | | | |
| --- | --- | --- | --- | --- | --- | --- |
|  | NC | | MCI | | AD | |
|  | PCC (95% CI) | p-value | PCC (95% CI) | p-value | PCC (95% CI) | p-value |
| brainageR | -0.03 (-0.09,0.04) | 0,415 | -0.23 (-0.28,-0.17) | <0.001 | -0.14 (-0.23,-0.04) | 0,006 |
| DeepBrainNet | 0.01 (-0.06,0.08) | 0,736 | -0.33 (-0.38,-0.27) | <0.001 | -0.24 (-0.33,-0.14) | <0.001 |
| brainage | -0.06 (-0.13,0.0) | 0,061 | -0.28 (-0.33,-0.22) | <0.001 | -0.26 (-0.35,-0.17) | <0.001 |
| ENIGMA | -0.05 (-0.12,0.01) | 0,125 | -0.28 (-0.33,-0.22) | <0.001 | -0.18 (-0.27,-0.08) | <0.001 |
| pyment | -0.02 (-0.09,0.04) | 0,496 | -0.23 (-0.29,-0.17) | <0.001 | -0.2 (-0.29,-0.11) | <0.001 |
| mccqrnn | -0.02 (-0.09,0.04) | 0,489 | -0.22 (-0.28,-0.16) | <0.001 | -0.1 (-0.2,-0.01) | 0,039 |
| Grey Matter | 0.05 (-0.02,0.11) | 0,161 | 0.19 (0.13,0.25) | <0.001 | 0.23 (0.14,0.32) | <0.001 |

1. Lövdén, M., Fratiglioni, L., Glymour, M. M., Lindenberger, U. & Tucker-Drob, E. M. Education and Cognitive Functioning Across the Life Span. *Psychol. Sci. Public Interest* **21**, 6–41 (2020).

2. Lipsitz, S. R., Leong, T., Ibrahim, J. & Lipshultz, S. A Partial Correlation Coefficient and Coefficient of Determination for Multivariate Normal Repeated Measures Data. *J. R. Stat. Soc. Ser. Stat.* **50**, 87–95 (2001).

| 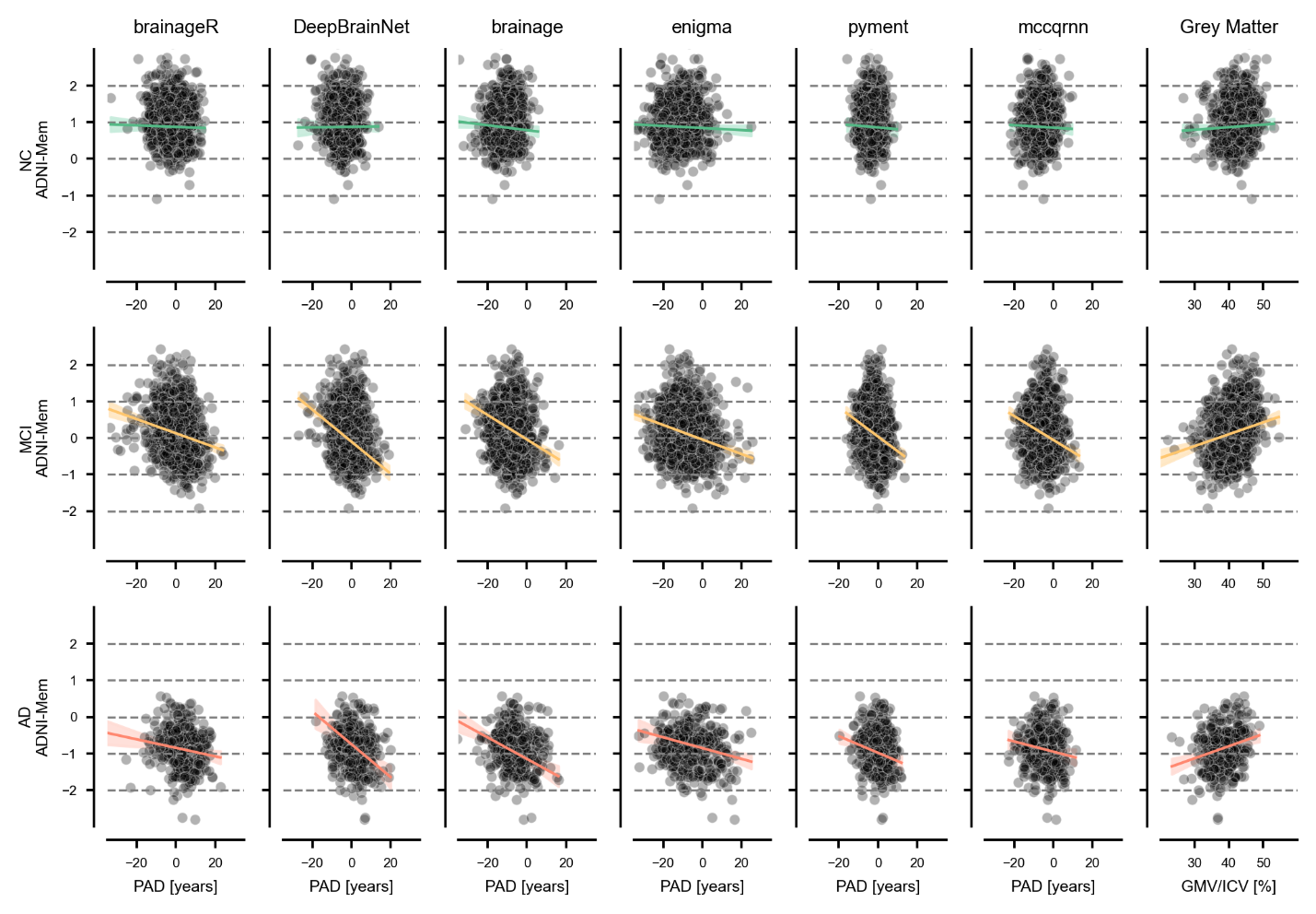 |
| --- |
| **Supplementary Figure 2:** Baseline PAD vs memory performance (ADNI-Mem) in NC, MCI, and AD. |

### Association between PAD and disease conversion

| 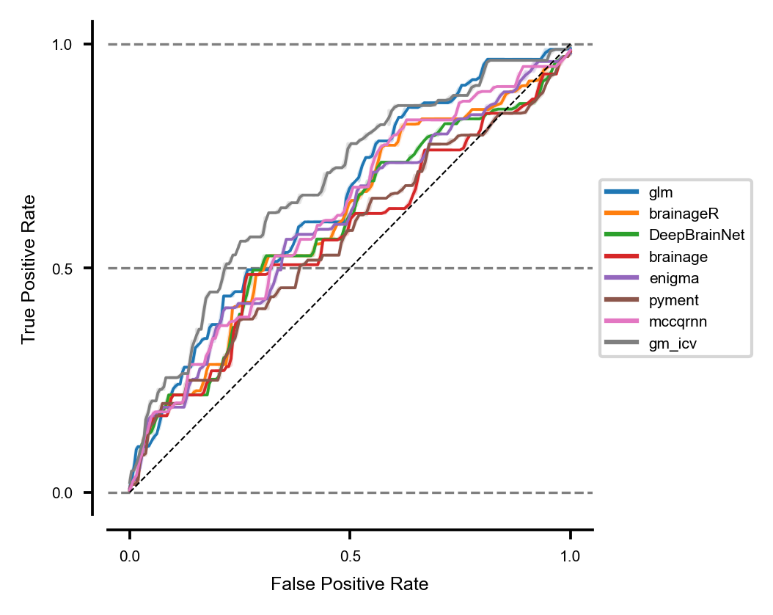 |
| --- |
| **Supplementary Figure 3:** Receiver operator curves for the logistic model to predict disease conversion for the six brain age models, normalised grey matter volume (gm_icv), and a model only including covariates (glm), i.e. no anatomical measure or predicted age deviation. |

| **Supplementary Table 4:** Association between PAD and longitudinal disease conversion from mild cognitive impairment (MCI) to Alzheimer’s disease (AD) within four years from baseline. The odds ratio and corresponding p-value are given for the coefficient. P(x) provides the probability of converting from MCI to AD for a hypothetical individual who has a PAD or grey matter volume (x) at 1 SD below (x = μ - SD) or above (x = μ+ SD) the mean at baseline. In addition, the Brier score and the AUC are provided as overall performance measures. In total, 275 out of 1083 subjects converted withing those four years. | | | | | | | |
| --- | --- | --- | --- | --- | --- | --- | --- |
| Model | Odds (95% CI) | p-value (Odds) | P(x= µ-SD) (95% CI) | P(x=µ)  (95% CI) | P(x= µ+SD)  (95% CI) | AUC  (95% CI) | Brier  (95% CI) |
| brainageR | 1.11 (1.07,1.16) | <0.001 | 0.25 (0.18,0.34) | 0.42 (0.35,0.49) | 0.61  (0.51,0.7) | 0.71  (0.65,0.78) | 0.2  (0.19,0.22) |
| DeepBrainNet | 1.27 (1.17,1.37) | <0.001 | 0.13 (0.07,0.22) | 0.36 (0.29,0.44) | 0.68 (0.58,0.77) | 0.77  (0.71,0.83) | 0.19 (0.17,0.21) |
| brainage | 1.3  (1.2,1.4) | <0.001 | 0.14 (0.08,0.22) | 0.44 (0.36,0.51) | 0.79  (0.69,0.87) | 0.77 (0.71,0.82) | 0.19  (0.16,0.21) |
| ENIGMA | 1.11 (1.07,1.15) | <0.001 | 0.24 (0.17,0.32) | 0.43 (0.36,0.5) | 0.64  (0.53,0.74) | 0.71  (0.65,0.77) | 0.2  (0.18,0.22) |
| pyment | 1.24 (1.15,1.33) | <0.001 | 0.21  (0.15,0.3) | 0.41 (0.34,0.48) | 0.65  (0.54,0.74) | 0.72  (0.66,0.78) | 0.2  (0.18,0.22) |
| mccqrnn | 1.22 (1.14,1.3) | <0.001 | 0.2  (0.14,0.29) | 0.42 (0.35,0.49) | 0.68  (0.58,0.76) | 0.73 (0.67,0.79) | 0.2  (0.18,0.22) |
| Grey Matter | 0.9 (0.8,1.01) | 0.063 | 0.54 (0.41,0.66) | 0.44  (0.37,0.5) | 0.34  (0.23,0.47) | 0.66  (0.6,0.73) | 0.22 (0.21,0.24) |

| 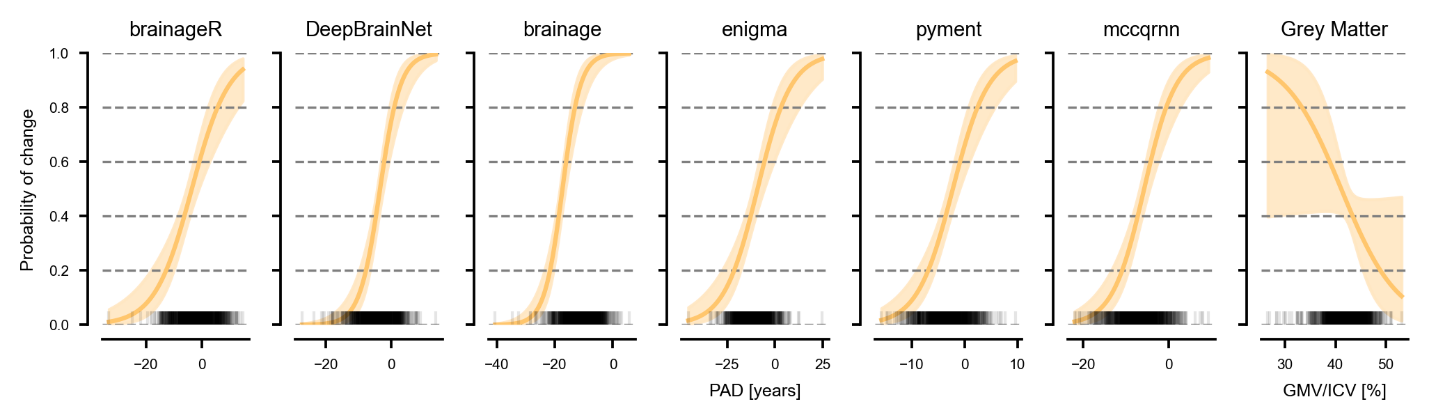 |
| --- |
| **Supplementary Figure 4:** The probability of conversion mild cognitive impairment (MCI) to Alzheimer’s disease (AD) plotted against baseline PAD values for each package. The black rug plot at the base of each subplot shows individual PAD values from the dataset. The curves are estimated with the covariates fixed to their respective means, and sex was set to female. The shaded areas represent the 95% confidence intervals. |

| **Supplementary Table 5:** Association between PAD and longitudinal disease conversion from normal cognition (NC) to mild cognitively impaired (MCI) or Alzheimer’s disease (AD) within four years from baseline. The hazard ratio (HR) and corresponding p-value are given for the coefficient. P(x) provides the cumulative hazard of converting from NC to MCI or AD for a hypothetical individual who has a PAD or grey matter volume (x) at 1 SD below (x = μ - SD) or above (x = μ+ SD) the mean at baseline. In addition, the Brier score and the AUC are provided as overall performance measures. In total, 275 out of 1083 subjects converted withing those four years. | | | | | | | |
| --- | --- | --- | --- | --- | --- | --- | --- |
| Model | HR (95% CI) | p-value (Hazard) | P(x= µ-SD) (95% CI) | P(x=µ)  (95% CI) | P(x= µ+SD)  (95% CI) | AUC  (95% CI) | Brier  (95% CI) |
| brainageR | 1.03 (0.99,1.08) | 0,127 | 0.11 (0.07,0.18) | 0.14  (0.09,0.2) | 0.17  (0.1,0.28) | 0.67  (0.58,0.76) | 0.12  (0.1,0.15) |
| DeepBrainNet | 1.09 (1.02,1.16) | 0,012 | 0.08 (0.04,0.16) | 0.12 (0.08,0.19) | 0.19  (0.12,0.31) | 0.69  (0.59,0.79) | 0.12  (0.09,0.15) |
| brainage | 1.11 (1.04,1.18) | 0,001 | 0.07 (0.04,0.12) | 0.12 (0.08,0.19) | 0.21  (0.13,0.34) | 0.73  (0.65,0.81) | 0.12  (0.09,0.14) |
| ENIGMA | 1.06 (1.02,1.09) | <0.001 | 0.09 (0.05,0.15) | 0.13  (0.09,0.2) | 0.2  (0.13,0.32) | 0.71  (0.62,0.79) | 0.12  (0.1,0.15) |
| pyment | 1.1 (1.03,1.18) | 0,007 | 0.09 (0.05,0.14) | 0.12 (0.08,0.18) | 0.18  (0.11,0.28) | 0.71  (0.63,0.79) | 0.12  (0.09,0.14) |
| mccqrnn | 1.01 (0.95,1.07) | 0,757 | 0.13  (0.08,0.2) | 0.13  (0.09,0.2) | 0.14  (0.08,0.24) | 0.66  (0.57,0.74) | 0.12  (0.1,0.15) |
| Grey Matter | 0.89 (0.83,0.95) | <0.001 | 0.21 (0.13,0.34) | 0.14 (0.09,0.21) | 0.09  (0.05,0.15) | 0.68  (0.59,0.77) | 0.12  (0.1,0.15) |

### Association between PAD and rate of decline in grey matter volume and memory function

| 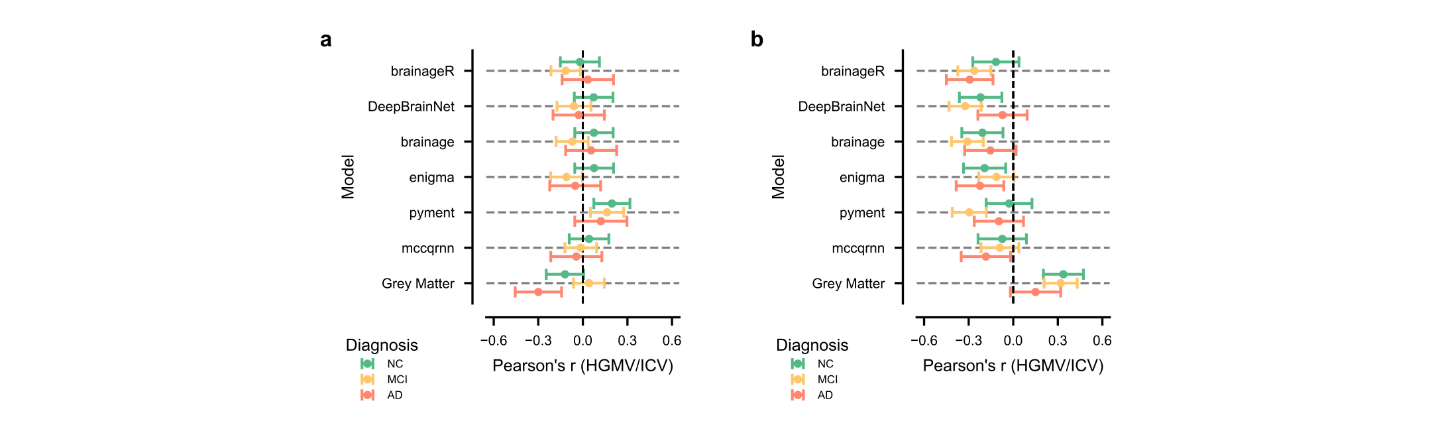 |
| --- |
| **Supplementary Figure 5:** Longitudinal analysis of PAD. **a)** The association between baseline PAD and four-year change in normalised hippocampal grey matter volume (HGMV/ICV). **b)** The association between four-year change in PAD and four-year change in normalised hippocampal grey matter volume (HGMV/ICV). |

| **Supplementary Table 6:** Association between the PAD at baseline and change in cognition or normalised grey matter volume within four years. Displayed are the correlation, 95% confidence interval (CI), and the corresponding p-value. The correlations were computed for each diagnostic group separately: normal cognition (NC), mild cognitive impairment (MCI), Alzheimer’s Disease (AD). | | | | | | | | | | | | |
| --- | --- | --- | --- | --- | --- | --- | --- | --- | --- | --- | --- | --- |
|  | **Adni-Mem** | | | | | | **GMV/ICV** | | | | | |
|  | AD | | NC | | MCI | | AD | | NC | | MCI | |
|  | r (95%) | p-value | r (95%) | p-value | r (95%) | p-value | r (95%) | p-value | r (95%) | p-value | r (95%) | p-value |
| **brainageR** | -0.33 (-0.48,-0.18) | <0.001 | -0.12 (-0.24,0.0) | 0,05 | -0.25 (-0.34,-0.16) | <0.001 | -0.27 (-0.45,-0.09) | 0,009 | -0.09 (-0.22,0.04) | 0,175 | -0.11 (-0.22,0.0) | 0,053 |
| **DeepBrainNet** | -0.4 (-0.54,-0.25) | <0.001 | 0.0 (-0.11,0.12) | 0,938 | -0.22 (-0.32,-0.12) | <0.001 | -0.21 (-0.39,-0.03) | 0,026 | 0.03 (-0.09,0.15) | 0,628 | -0.05 (-0.18,0.08) | 0,435 |
| **brainage** | -0.34 (-0.49,-0.19) | <0.001 | -0.05 (-0.17,0.07) | 0,397 | -0.3 (-0.39,-0.21) | <0.001 | -0.17 (-0.35,0.0) | 0,053 | -0.07 (-0.19,0.05) | 0,262 | -0.07 (-0.19,0.05) | 0,272 |
| **enigma** | -0.24 (-0.4,-0.08) | 0,005 | -0.06 (-0.18,0.06) | 0,305 | -0.27 (-0.37,-0.18) | <0.001 | -0.25 (-0.42,-0.08) | 0,008 | 0.06 (-0.07,0.18) | 0,344 | -0.07 (-0.19,0.05) | 0,247 |
| **pyment** | -0.36 (-0.51,-0.21) | <0.001 | -0.01 (-0.13,0.1) | 0,851 | -0.21 (-0.31,-0.11) | <0.001 | -0.08 (-0.27,0.12) | 0,389 | 0.03 (-0.09,0.15) | 0,606 | 0.08 (-0.05,0.21) | 0,229 |
| **mccqrnn** | -0.3 (-0.46,-0.15) | <0.001 | 0.02 (-0.1,0.15) | 0,701 | -0.15 (-0.24,-0.05) | 0,003 | -0.13 (-0.31,0.05) | 0,129 | 0.03 (-0.1,0.16) | 0,644 | -0.04 (-0.16,0.08) | 0,476 |
| **Grey Matter** | 0.06 (-0.11,0.22) | 0,493 | 0.09 (-0.03,0.2) | 0,158 | 0.25 (0.16,0.34) | <0.001 | -0.24 (-0.4,-0.09) | 0,003 | 0.03 (-0.09,0.15) | 0,595 | 0.12 (-0.0,0.24) | 0,054 |

| **Supplementary Table 7:** Association between the PAD at baseline and change in normalised hippocampal grey matter volume within four years. Displayed are the correlation, 95% confidence interval (CI), and the corresponding p-value. The correlations were computed for each diagnostic group separately: normal cognition (NC), mild cognitive impairment (MCI), Alzheimer’s Disease (AD). | | | | | | |
| --- | --- | --- | --- | --- | --- | --- |
|  | **Hippocampus GMV / ICV** | | | | | |
|  | AD | | NC | | MCI | |
|  | r (95%) | p-value | r (95%) | p-value | r (95%) | p-value |
| **brainageR** | 0.03 (-0.14,0.21) | 0,689 | -0.02 (-0.15,0.11) | 0,76 | -0.12 (-0.21,-0.02) | 0,024 |
| **DeepBrainNet** | -0.03 (-0.2,0.14) | 0,737 | 0.07 (-0.06,0.2) | 0,266 | -0.06 (-0.17,0.05) | 0,299 |
| **brainage** | 0.06 (-0.12,0.23) | 0,505 | 0.07 (-0.05,0.2) | 0,249 | -0.07 (-0.18,0.04) | 0,189 |
| **enigma** | -0.05 (-0.22,0.12) | 0,536 | 0.08 (-0.05,0.21) | 0,246 | -0.11 (-0.22,-0.0) | 0,043 |
| **pyment** | 0.12 (-0.05,0.3) | 0,16 | 0.2 (0.07,0.32) | 0,003 | 0.16 (0.05,0.27) | 0,005 |
| **mccqrnn** | -0.04 (-0.22,0.13) | 0,598 | 0.04 (-0.09,0.18) | 0,53 | -0.01 (-0.12,0.09) | 0,79 |
| **Grey Matter** | -0.3 (-0.46,-0.14) | 0,001 | -0.12 (-0.25,0.01) | 0,061 | 0.04 (-0.06,0.14) | 0,432 |

### Association between changes in the PAD and decline in grey matter volume and memory function

| **Supplementary Table 8:** Association between the change in the PAD and change in cognition or normalised grey matter volume within four years. Displayed are the correlation, 95% confidence interval (CI), and the corresponding p-value. The correlations were computed for each diagnostic group separately: normal cognition (NC), mild cognitive impairment (MCI), Alzheimer’s Disease (AD). Change values were controlled for their respective baseline values. | | | | | | | | | | | | |
| --- | --- | --- | --- | --- | --- | --- | --- | --- | --- | --- | --- | --- |
|  | **Change in Adni-Mem** | | | | | | **Change in GMV/ICV** | | | | | |
|  | AD | | NC | | MCI | | AD | | NC | | MCI | |
|  | r (95%) | p-value | r (95%) | p-value | r (95%) | p-value | r (95%) | p-value | r (95%) | p-value | r (95%) | p-value |
| **brainageR** | -0.05 (-0.22,0.12) | 0,521 | 0.02 (-0.12,0.17) | 0,744 | 0.03 (-0.1,0.15) | 0,672 | -0.13 (-0.3,0.04) | 0,119 | 0.07 (-0.1,0.24) | 0,4 | 0.07 (-0.05,0.19) | 0,252 |
| **DeepBrainNet** | 0.03 (-0.13,0.2) | 0,683 | -0.1 (-0.24,0.04) | 0,173 | -0.15 (-0.26,-0.03) | 0,017 | 0.15 (-0.02,0.32) | 0,081 | -0.19 (-0.33,-0.04) | 0,015 | -0.28 (-0.39,-0.16) | <0.001 |
| **brainage** | -0.28 (-0.44,-0.12) | 0,001 | -0.12 (-0.26,0.02) | 0,081 | -0.24 (-0.34,-0.13) | <0.001 | -0.22 (-0.39,-0.06) | 0,012 | -0.19 (-0.34,-0.05) | 0,011 | -0.28 (-0.4,-0.16) | <0.001 |
| **enigma** | -0.09 (-0.26,0.07) | 0,262 | 0.09 (-0.04,0.23) | 0,169 | -0.0 (-0.12,0.12) | 0,986 | -0.03 (-0.2,0.14) | 0,725 | -0.13 (-0.28,0.02) | 0,093 | -0.06 (-0.19,0.07) | 0,375 |
| **pyment** | -0.29 (-0.44,-0.14) | 0,001 | -0.22 (-0.36,-0.08) | 0,002 | -0.44 (-0.54,-0.35) | <0.001 | -0.23 (-0.39,-0.06) | 0,009 | -0.31 (-0.45,-0.18) | <0.001 | -0.27 (-0.39,-0.14) | <0.001 |
| **mccqrnn** | -0.12 (-0.29,0.04) | 0,134 | -0.01 (-0.16,0.15) | 0,932 | -0.1 (-0.22,0.02) | 0,113 | -0.01 (-0.18,0.17) | 0,935 | -0.02 (-0.19,0.15) | 0,808 | -0.18 (-0.32,-0.04) | 0,013 |
| **Grey Matter** | 0.36 (0.21,0.51) | <0.001 | 0.25 (0.11,0.38) | 0,001 | 0.29 (0.17,0.42) | <0.001 |  |  |  |  |  |  |

| **Supplementary Table 9:** Unadjusted association between the change in the PAD and change in cognition or normalised grey matter volume within four years. Displayed are the correlation, 95% confidence interval (CI), and the corresponding p-value. The correlations were computed for each diagnostic group separately: normal cognition (NC), mild cognitive impairment (MCI), Alzheimer’s Disease (AD). | | | | | | | | | | | | |
| --- | --- | --- | --- | --- | --- | --- | --- | --- | --- | --- | --- | --- |
|  | **Change in Adni-Mem** | | | | | | **Change in GMV/ICV** | | | | | |
|  | AD | | NC | | MCI | | AD | | NC | | MCI | |
|  | r (95%) | p-value | r (95%) | p-value | r (95%) | p-value | r (95%) | p-value | r (95%) | p-value | r (95%) | p-value |
| **brainageR** | 0.05 (-0.09,0.19) | 0,454 | 0.06 (-0.06,0.19) | 0,323 | -0.06 (-0.22,0.11) | 0,465 | 0.09 (-0.07,0.26) | 0,249 | 0.12 (-0.0,0.24) | 0,052 | -0.21 (-0.37,-0.05) | 0,013 |
| **DeepBrainNet** | -0.09 (-0.23,0.04) | 0,177 | -0.17 (-0.28,-0.05) | 0,006 | 0.01 (-0.15,0.18) | 0,866 | -0.19 (-0.33,-0.04) | 0,013 | -0.24 (-0.36,-0.13) | <0.001 | 0.09 (-0.08,0.26) | 0,28 |
| **brainage** | -0.09 (-0.22,0.05) | 0,189 | -0.21 (-0.32,-0.1) | <0.001 | -0.3 (-0.46,-0.15) | 0,001 | -0.16 (-0.3,-0.01) | 0,031 | -0.22 (-0.34,-0.1) | <0.001 | -0.3 (-0.46,-0.15) | 0,001 |
| **enigma** | 0.1 (-0.04,0.23) | 0,148 | 0.0 (-0.12,0.12) | 0,978 | -0.13 (-0.29,0.03) | 0,104 | -0.13 (-0.28,0.02) | 0,082 | -0.02 (-0.15,0.11) | 0,724 | -0.1 (-0.27,0.07) | 0,255 |
| **pyment** | -0.22 (-0.36,-0.09) | 0,002 | -0.52 (-0.61,-0.43) | <0.001 | -0.3 (-0.45,-0.16) | <0.001 | -0.32 (-0.46,-0.18) | <0.001 | -0.27 (-0.4,-0.14) | <0.001 | -0.24 (-0.4,-0.08) | 0,006 |
| **mccqrnn** | -0.02 (-0.17,0.14) | 0,828 | -0.12 (-0.24,0.01) | 0,062 | -0.13 (-0.3,0.03) | 0,098 | -0.03 (-0.19,0.14) | 0,761 | -0.17 (-0.3,-0.03) | 0,019 | -0.05 (-0.22,0.12) | 0,565 |
| **Grey Matter** | 0.25 (0.12,0.38) | 0,001 | 0.37 (0.25,0.48) | <0.001 | 0.31 (0.15,0.46) | 0,001 | - | - | - | - | - | - |

| 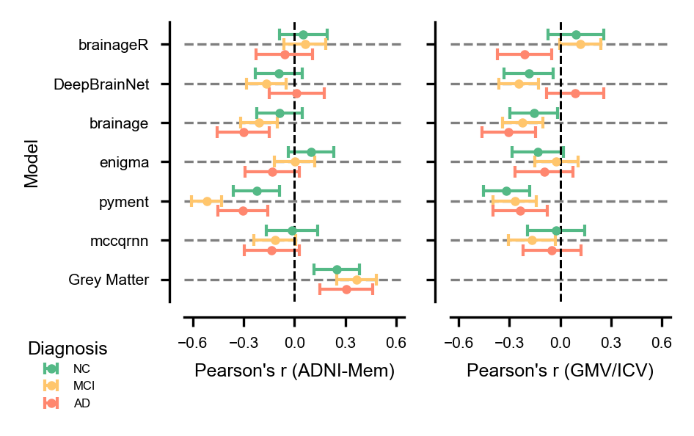 |
| --- |
| **Supplementary Figure 6:** Longitudinal analysis of PAD. The unadjusted association between four-year change in PAD and four-year change in normalised grey matter volume (GMV/ICV) or memory performance (ADNI-Mem). |

| **Supplementary Table 10:** Association between the change in the PAD and change in normalised hippocampal grey matter volume within four years. Displayed are the correlation, 95% confidence interval (CI), and the corresponding p-value. The correlations were computed for each diagnostic group separately: normal cognition (NC), mild cognitive impairment (MCI), Alzheimer’s Disease (AD). | | | | | | |
| --- | --- | --- | --- | --- | --- | --- |
|  | **Hippocampus GMV / ICV** | | | | | |
|  | AD | | NC | | MCI | |
|  | r (95%) | p-value | r (95%) | p-value | r (95%) | p-value |
| **brainageR** | -0.29 (-0.45,-0.14) | 0,001 | -0.12 (-0.27,0.04) | 0,132 | -0.26 (-0.37,-0.15) | <0.001 |
| **DeepBrainNet** | -0.07 (-0.24,0.09) | 0,371 | -0.22 (-0.36,-0.08) | 0,004 | -0.32 (-0.43,-0.22) | <0.001 |
| **brainage** | -0.15 (-0.33,0.02) | 0,078 | -0.21 (-0.35,-0.07) | 0,005 | -0.31 (-0.42,-0.2) | <0.001 |
| **enigma** | -0.22 (-0.38,-0.06) | 0,009 | -0.19 (-0.33,-0.05) | 0,01 | -0.11 (-0.23,0.0) | 0,059 |
| **pyment** | -0.1 (-0.26,0.07) | 0,234 | -0.03 (-0.18,0.13) | 0,71 | -0.3 (-0.41,-0.18) | <0.001 |
| **mccqrnn** | -0.18 (-0.35,-0.02) | 0,032 | -0.07 (-0.24,0.09) | 0,357 | -0.09 (-0.22,0.04) | 0,161 |
| **Grey Matter** | 0.15 (-0.02,0.32) | 0,079 | 0.34 (0.2,0.47) | <0.001 | 0.32 (0.21,0.43) | <0.001 |

| 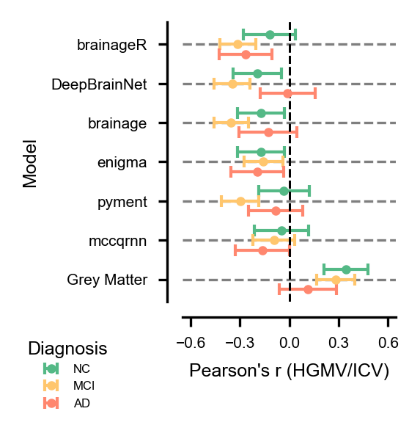 |
| --- |
| **Supplementary Figure 7:** The association between four-year change in PAD and four-year change in normalised hippocampal grey matter volume (HGMV/ICV), both adjusted for their respective baseline values. |

| **Supplementary Table 11:** Adjusted association between the change in the PAD and change in normalised hippocampal grey matter volume within four yearsDisplayed are the correlation, 95% confidence interval (CI), and the corresponding p-value. The correlations were computed for each diagnostic group separately: normal cognition (NC), mild cognitive impairment (MCI), Alzheimer’s Disease (AD). | | | | | | |
| --- | --- | --- | --- | --- | --- | --- |
|  | **Hippocampus GMV / ICV** | | | | | |
|  | AD | | NC | | MCI | |
|  | r (95%) | p-value | r (95%) | p-value | r (95%) | p-value |
| **brainageR** | -0.26 (-0.42,-0.1) | 0,003 | -0.12 (-0.28,0.04) | 0,122 | -0.31 (-0.42,-0.2) | <0.001 |
| **DeepBrainNet** | -0.01 (-0.18,0.16) | 0,887 | -0.2 (-0.34,-0.05) | 0,01 | -0.35 (-0.46,-0.24) | <0.001 |
| **brainage** | -0.13 (-0.3,0.05) | 0,137 | -0.17 (-0.32,-0.03) | 0,019 | -0.35 (-0.46,-0.25) | <0.001 |
| **enigma** | -0.19 (-0.36,-0.03) | 0,021 | -0.17 (-0.32,-0.03) | 0,02 | -0.16 (-0.28,-0.04) | 0,011 |
| **pyment** | -0.08 (-0.25,0.08) | 0,306 | -0.03 (-0.19,0.12) | 0,658 | -0.3 (-0.41,-0.18) | <0.001 |
| **mccqrnn** | -0.16 (-0.33,0.0) | 0,054 | -0.05 (-0.21,0.12) | 0,543 | -0.09 (-0.22,0.03) | 0,146 |
| **Grey Matter** | 0.11 (-0.06,0.29) | 0,187 | 0.35 (0.21,0.48) | <0.001 | 0.28 (0.16,0.4) | <0.001 |
